# Exosomes as Crucial Players in Pathogenesis of Systemic Lupus Erythematosus

**DOI:** 10.1155/2022/8286498

**Published:** 2022-07-20

**Authors:** Yue Fei, Qi Liu, Na Peng, Guocan Yang, Ziwei Shen, Pan Hong, Shengjun Wang, Ke Rui, Dawei Cui

**Affiliations:** ^1^Shaoxing University School of Medicine, Shaoxing, China; ^2^Department of Hematology, The First Affiliated Hospital of Shaoxing University, Shaoxing, China; ^3^Department of Transfusion, Shaoxing People's Hospital (Shaoxing Hospital, Zhejiang University School of Medicine), Shaoxing, China; ^4^Department of Rheumatology and Nephrology, The Second People's Hospital, China Three Gorges University, Yichang, China; ^5^Department of Laboratory Medicine, Affiliated Hospital of Jiangsu University, Zhenjiang, China; ^6^Department of Hematology, Shaoxing People's Hospital (Shaoxing Hospital, Zhejiang University School of Medicine), Shaoxing, China; ^7^Institute of Laboratory Medicine, Jiangsu Key Laboratory for Laboratory Medicine, Jiangsu University School of Medicine, Zhenjiang, China; ^8^Department of Laboratory Medicine, Affiliated People's Hospital, Jiangsu University, Zhenjiang, China; ^9^Department of Blood Transfusion, The First Affiliated Hospital, Zhejiang University School of Medicine, Hangzhou, China

## Abstract

Systemic lupus erythematosus (SLE) is a systemic autoimmune disease that affects multiple systems. Its clinical manifestation varies across patients, from skin mucosa to multiorgan damage to severe central nervous system involvement. The exosome has been shown to play an important role in the pathogenesis of autoimmune diseases, including SLE. We review the recent knowledge of exosomes, including their biology, functions, mechanism, and standardized extraction and purification methods in SLE, to highlight potential therapeutic targets for SLE.

## 1. Introduction

Systemic lupus erythematosus (SLE) is a chronic, systemic, and severe autoimmune disease that affects multiple systems. Patients with SLE have a poor quality of life and high mortality [1] and are more likely to develop comorbidities such as cardiovascular and respiratory diseases, infections, cancers, and osteoporosis [2–5]. Moreover, women are more likely to suffer from SLE than men [6]. Currently, SLE is treated with the application of biological agents, which provide relief and minimize the use of glucocorticoids [7]. SLE patients still require long-term drug-based maintenance, which often has toxic side effects [8]. Long-term use of glucocorticoids can even lead to emotional disorders such as depression in patients [9].

Exosomes as a targeted carrier may reduce drug concentrations in the human body and the accumulation of drug toxicity [10]. Many mechanisms are involved in the etiology and pathogenesis of SLE, but these remain unclear. Exosomes play an important role in innate and adaptive immunity, participate in many physiological and pathological SLE processes, and help maintain immune homeostasis [11]. In recent years, the effect of exosomes in SLE has attracted greater attention. This review introduces exosomes, their immunomodulatory role and mechanism, and their potential as a new SLE drug target and identifies new opportunities for understanding SLE pathogenesis and biotherapy.

## 2. Exosome Classification

Extracellular vesicles (EVs) are membrane-derived vesicles surrounded by lipid bilayers in the periphery that are released into the extracellular space by various cell types, mediate intercellular communication, and can be found in various bodily fluids [12]. EVs can be classified based on their release processes as microvesicles (MVs), exosomes, and apoptotic bodies (APBs) [13]. MVs are produced by budding directly from the cell membrane to outside the cell [14], and APBs arise as part of the apoptotic process [15]. Multivesicular bodies (MVBs) are late endosomes that fuse with cell membranes and release their contents as exosomes [16].

EVs are vesicles 30 to 1000 nm or more in size [17]. Exosomes are one type of EV with a size of 30 to 150 nm [18] that contain many transmembrane proteins, including CD9, CD63, and CD81 [19]. Tetraspanin proteins are abundant in the outer membrane and can indirectly control cell interactions through exosomes. They play important roles in regulating physiological processes such as signal transduction, motility, adhesion, cell activation, and tissue differentiation [20]. CD63 is mainly found in MVBs and lysosomes and is closely related to exosome production [21]. Studies have suggested that CD63 is the defining exosome transmembrane protein [22]. The quantification and detection of CD63 on EVs by nanoflow cytometry can determine exosome content in body fluids [23]. CD9 is also present in the endosome system, particularly in MVBs, where it is located on the cell surface and facilitates the endocytosis of CD9-positive exosomes [24]. Therefore, CD9 can promote intercellular exosome transport. In addition, studies have suggested that high levels of CD9 on the plasma membrane may be associated with early endosome formation, while CD63 mainly affects the MVB stage [25]. CD9 and CD63 may be associated with exosome formation. The CD29/CD81 complex on the cell surface also promotes intercellular exosome transport [26].

**Figure 1:**
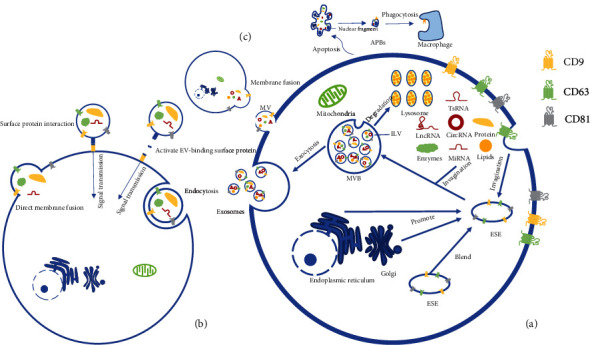
Biogenesis, secretion, and ingestion of EVs. (a) Exosomes from ILV in MVBs are secreted outside cells by exocytosis, transporting lipids, lncRNA, miRNA, circRNA, proteins, tsRNA, and enzymes between cells. CD63 is a defining exosome transmembrane protein. (b) Exosomes act in multiple ways on receptor cells. (c) Origin and secretion of MV and APBs. Key: APBs: apoptotic bodies; ESE: early-sorting endosome; ILV: intraluminal vesicles; MV: microvesicles; MVB: multivesicular bodies; tsRNA: tRNA-derived small RNAs.

EVs are exosomes surrounded by lipid bilayers that are released by various cells, including macrophages, dendritic cells (DCs), tumor cells, and mesenchymal stem cells (MSCs) [27]. Exosome formation is mainly dependent on the double invagination of the plasma membrane. In the first exosome invagination, the plasma membrane envelopes soluble proteins in the extracellular environment, gradually forming an early-sorting endosome (ESE). The trans-Golgi network and endoplasmic reticulum also facilitate the forming and increasing of ESE content [28, 29]. ESEs can also fuse and eventually mature into late-sorting endosomes (LSEs). With the second exosome invagination, MVBs containing multiple intracavitary vesicles (ILVs) begin to form. The fusion of MVBs with the plasma membrane releases ILVs that become exosomes in the extracellular fluid [30] or are degraded via fusion with lysosomes (Figure 1) [31].

**Figure 2:**
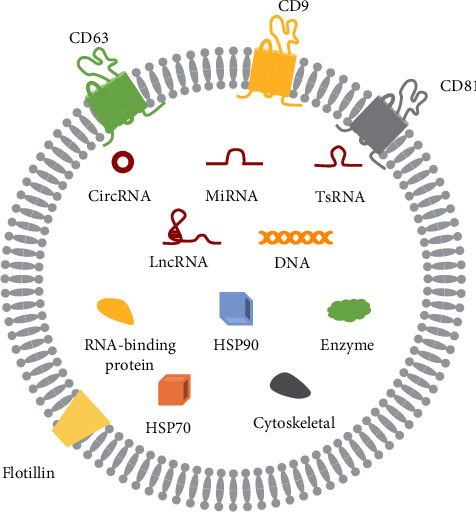
The structure and contents of exosomes. Exosome is a kind of vesicle surrounded by a lipid bilayer in the periphery, and it contains nucleic acid, lipid, protein, and other substances. HSP70: heat shock protein 70; HSP90: heat shock protein 90; tsRNA: tRNA-derived small RNAs.

Exosomes are widely distributed in various body fluids [32] and contain adhesion molecules, tetrads, enzymes, scaffolds, nucleic acids, and binding proteins (Figure 2) [33]. Nucleic acids, lipids, and proteins can be transferred between cells via exosomes [34], in some cases affecting recipient cells via autocrine and paracrine mechanisms [35]. There are multiple modes of action between target cells and exosomes. The exosome information transmission process can occur on the cell surface. Exosomes and cells can simply transmit information through receptor-ligand interaction, or EV surface proteins can be activated without entering the cell [36, 37]. Other modes of action include direct membrane fusion and endocytosis, which includes receptor-mediated endocytosis, phagocytosis, and macropinocytosis [38–42].

## 3. Exosome Function

The functions of exosomes from other cells differ according to the substances they are carrying. For example, macrophage-derived exosomes can overexpress ArfGAP with GTPase domain ankyrin repeat and PH domain 2 (*AGAP2*) antisense RNA 1 (*AGAP2-AS1*) or underexpress microRNA- (miRNA-) 296 (*miR-296*) to enhance the antiradiotherapy capability of lung cancer cells [43]. Similarly, exosomes derived from M2 macrophages use apolipoprotein E (ApoE), a lipid-transporting lipoprotein found within the brain and periphery, to promote gastric cancer cell migration [44]. In addition, lung adenocarcinoma (LUAD) cells acquire enhanced cell migration, invasion, and angiogenic abilities by absorbing M2 macrophage-derived exosomes [45]. Furthermore, mature DC exosomes can promote osteogenic differentiation and improve bone regeneration by transporting miRNA-335 (miR-335) in thighbone-deficient thymic rats [46]. Moreover, tumor-derived exosomes can promote the polarization of M2 macrophages, while exosomes carrying miRNA-19b-3p (miR-19b-3p) can promote lung cancer metastasis via the Hippo pathway [47]. Finally, exosomes from hepatocellular carcinoma (HCC) cells can promote tumorigenesis by secreting sonic hedgehog (Shh) protein [48], which is closely related to both embryonic development and histogenesis in mammals. These examples highlight how exosome functions are closely related to their origin and contents.

## 4. Exosome Features

Exosomes are small, can avoid phagocytosis by mononuclear macrophages, and can freely cross the vessel wall and extracellular matrix [49]. Exosomes carry molecules such as CD55 and CD59 on their surface, preventing their damage by complement or coagulation factors [50]. Therefore, CD55 and CD59 can maintain exosome stability. As intercellular transport vesicles, exosomes have remarkable properties, including not stimulating the immune system, avoiding degradation, carrying endogenous bioactive molecules, long persistence, and crossing multiple biological barriers [51, 52]. Small molecule drugs, including functional nucleic acid nanoparticles, may be incorporated into and carried by exosomes [53, 54]. Exosomes have a high degree of biological stability and can stably exist in the blood for an extended time [55]. In addition, the exosome's specific molecular surface structure can be used to target specific cells [56]. Therefore, exosomes represent a suitable carrier in drug delivery systems.

## 5. Exosomes as Potential Biomarkers

Studies have found that some miRNAs can be used to diagnose lupus nephritis (LN) based on their levels in urine-borne exosomes of SLE patients [57, 58] and as predictors of early fibrosis [59–61] and the need for LN treatment [62]. In addition, S100 calcium-binding protein A4 (S100A4) protein levels can be used for evaluating LN activity [63]. T cell-derived exosomes contain many molecules, including miRNAs, long noncoding RNAs (lncRNAs), circular RNAs (circRNAs), S100A4, ApoE, and bactericidal permeability-increasing protein (BPI), which can be transported between cells. Therefore, exosomes can be used as novel biomarkers and predictors of SLE progression (Table 1). However, the dearth of highly sensitive exosome detection methods limits their use as potential SLE biomarkers. Cascade signal amplification is one such method that has been proposed based on a biosensor able to detect exosomes at concentrations as low as 44 particles/*μ*L [64]. Alternatively, exosomes can be detected using a human CD63 antibody conjugated to a molecule that enhances the fluorescence of the Alexa Fluor 647 (AF647) dye [65]. These highly sensitive and specific methods for detecting exosomes in the body fluids of patients offer potential diagnostic approaches for SLE biomarkers.

## 6. Exosome Regulation in SLE

### 6.1. Negative Regulation of Exosomes in SLE

Exosomes have different effects on recipient cells based on their different sources and substances carried [66]. MSCs are pluripotent stem cells with the ability for self-renewal and multidirectional differentiation. Previous studies have shown that MSCs alleviate LN by inhibiting T follicular helper (Tfh) cell development and subsequent humoral immune activation [67]. MSC-derived exosomes (MSC-Exos) have similar functions to MSCs in treating autoimmune diseases, such as repairing damaged tissue, regulating the immune response, and playing an anti-inflammatory role. While increases in MSC-Exos or their inhibitory function may be beneficial for treating autoimmune diseases, they may improve the immunity of tumors and chronic infectious pathogens. SLE is a chronic autoimmune disease caused by the production of various autoantibodies that can affect and damage multiple organs and systems [68].

The initial stage of SLE is macrophage activation [69]. Macrophages participate in immune and inflammatory processes and acquire different polarized phenotypes in these processes or responses. The polarized macrophage phenotype includes classically activated macrophages (M1) and selectively activated macrophages (M2) [70]. M1 macrophages are closely associated with SLE development and aggression, while M2 macrophages can reduce SLE severity [71]. However, MSC-Exos can inhibit the M1 macrophage polarization, but its mechanism is imprecise. MSC-Exos can increase transfer RNA- (tRNA-) derived small RNA (tsRNA) 21109 (*tsRNA-21109*) expression, affecting Rap, Ras, Hippo, Wnt, mitogen-activated protein kinase (MAPK), and transforming growth factor *β* (TGF*β*) signaling pathway and inhibiting the immune response, leading to decreased M1 and increased M2 activity [72].

MSC-Exos have immunosuppressive effects on B lymphocytes [73, 74] and regulate the T helper (Th) and regulatory (Treg) cell subgroups to reduce the cytotoxicity and proliferation of cytotoxic T cells and the inflammatory response in SLE patients [75, 76]. It has been reported that MSC-EVs isolated from adipose tissues can improve the structure and function of the kidney and reduce kidney damage and dysfunction by upregulating interleukin 10 (IL-10) expression in a new porcine model of metabolic syndrome (METS) and renal artery stenosis (RAS) [77]. In addition, a study has shown that the direct injection of human bone marrow mesenchymal stem cells into mice with LN helps to control inflammation [78]. However, no studies have yet explored the use of MSC-EVs for treating SLE in mice or humans. Nevertheless, exosomes derived from professional antigen-presenting cells (APCs) can regulate the immune response, and DC-derived EVs (DC-EVs) have been found to have the same effect as DC cells in treating autoimmune diseases [79].

### 6.2. Positive Regulation of Exosomes in SLE

T cell-derived exosomes have the opposite effect as those from MSCs. These exosomes were found to cause chronic immune activation and produce excessive cytokines and chemokines via the relationship of cell subgroups with lupus type I interferon (IFN) signaling [80]. In addition, exosome delivery of miRNAs promotes IFN*α* secretion by human plasmacytoid DCs (pDCs) via Toll-like receptor 7 (TLR7) [81]. IFN is one of the most critical cytokines in SLE [82], promoting SLE progression by affecting CD8^+^ T cells in patients [83]. Increased serum IFN in SLE patients has been found to negatively correlate with component 3 (C3) and 4 (C4) levels [84]. IFN can interact with pDC, T cells, B cells, natural killer (NK) cells, and macrophages to increase their survival and maturation [85]. Inflammatory cytokine and chemokine levels are elevated in SLE patients with elevated IFN levels [80], who are also more likely to develop LN and have a poorer response to immunosuppressive treatment [86]. Therefore, T cell-derived exosomes can promote autoimmunity via cytokines such as IFN.

Studies have suggested that a lack of S100A4 in exosomes derived from highly metastatic HCC (HMH) reduces tumor necrosis factor *α* (*TNFα*) expression in the mouse [87, 88], indicating that S100A4 acts to increase TNF*α* levels. Soluble S100A4 can directly activate the protein kinase B (Akt) signaling pathway to prolong CD8^+^ T cell survival [89] and promote SLE development [90, 91] through the ability of CD8^+^ T cells to create autoantibodies and cause organ damage [92]. HMH-derived exosomes were found to have an adverse effect on SLE in patients. Therefore, blocking exosome secretion or inhibiting the production of related pathogenic carriers may be beneficial in treating SLE.

## 7. Exosomes in SLE treatment

Exosomes have unique benefits compared to other carriers in SLE treatment. Exosomes have a long half-life, existing for extended periods in the body [93] and can be stored for long periods, either for short periods at 4°C and -20°C or for long periods at -80°C [94]. Exosomes can transport proteins and nucleic acids between cells, protecting them from degradation when they enter cells [95, 96]. Exosomes are small enough to cross biological membranes and even have the capacity to cross biological barriers such as the blood-brain barrier (BBB) and blood-cerebrospinal fluid barrier (BCSFB) [97]. Exosomes can carry different drugs to meet treatment needs [98], prolonging the drug's half-life and increasing the stability of its release [99]. As drug carriers, exosomes have the innate advantages of prolonged stability, convenient storage, content protection, avoiding immune monitoring, and crossing biological barriers. Therefore, exosomes have the potential to play a more significant role in SLE therapies.

SLE treatment is aimed at alleviating symptoms, preventing damage accumulation, and minimizing drug side effects, improving patients' long-term prognosis and quality of life. Immunotherapy for patients with autoimmune diseases usually lasts for their whole life. The continuous use of drugs can produce severe adverse reactions and side effects. In recent years, treatment options for SLE patients have been continually updated. Hydroxychloroquine (HCQ) is commonly prescribed for SLE treatment at a dose of no more than 5 mg/kg. Glucocorticoid (GC) doses should be reduced to <7.5 mg during chronic maintenance treatment and eliminated when possible, and the appropriate use of immunomodulators such as methotrexate, azathioprine, and mycophenolate can accelerate the gradual reduction and discontinuation of GC. The addition of belimumab should be considered for persistently active or flaring extrarenal disease. Rituximab (RTX) is recommended for various organ-threatening, refractory conditions [100]. Early treatment can effectively stop disease progression and improve the patients' long-term quality of life.

Exosome-based drug delivery has been widely reported. The ideal therapeutic strategy is to reduce the required drug concentrations via their targeted delivery, preventing damage accumulation and minimizing side effects. Studies have designed various experimental methods for injecting specific drugs into exosomes and achieving targeted exosome-based therapy. The increasing understanding and development of therapeutic nucleic acids (TNA) [101] enabled plasmid DNA (pDNA) encoding the anti-inflammatory cytokine interleukin 10 (IL-10 pDNA) and the chemotherapeutic drug betamethasone sodium phosphate (BSP) to be incorporated into M2 macrophage-derived exosomes. The results showed that the molecules carried by the exosomes accumulated in large amounts at the target site within the mouse and had beneficial effects [102], indicating that modified exosomes show efficacy in treating autoimmune diseases [103]. However, there were practical problems in this study associated with how to safely manufacture and ensure the quality of exosomes. Nevertheless, delivering drugs to targeted sites via modified exosomes may represent a promising new approach for treating SLE. However, whether it can be safely applied in humans and how to modify exosomes for SLE are complex problems that remain to be solved.

## 8. Limitations of Exosomes in SLE Treatment

While exosomes have excellent prospects as drug carriers, they also have limitations. The first issue is how to extract and purify the exosomes. Currently, the common method for exosome separation requires ultrafiltration, immunoaffinity, and ultracentrifugation [104]. Differential ultracentrifugation remains the gold standard for exosome separation, but it causes mechanical damage to exosomes and is very time-consuming [105]. The recent development of cutting-edge biosensors for exosome detection and analysis has attracted significant attention because of their speed, convenience, low sample requirements, and high sensitivity and specificity, enabling significant progress in exosome separation and detection [106–108]. Biosensor-based detection and analysis were found to be much better than the traditional methods [109] and may accelerate the study of exosomes to treat SLE. However, due to the unique physical and chemical properties of protein molecules and the lack of exosome classification for transport, it remains difficult to inject them into exosomes. Nevertheless, a new type of engineered exosome has been reported into which therapeutic membrane proteins and soluble protein cargo can be injected [110]. Therefore, resolving these issues has made exosome-based drug delivery to target cells possible. However, the characteristics of exosomes alone were not sufficient to achieve the targeted transport of exogenous cargo to the target tissues. Relevant engineering technologies still under development will be required to improve exome targeting [111–113].

## 9. Conclusion

Exosomes play important roles in SLE occurrence and development through various molecular mechanisms that significantly mediate its progression. Through continuous research on exosomes, it may be possible to deliver drugs for long-term use with low side effects for treating SLE. Exosomes have attracted increasing attention from pharmacologists and drug developers as potential drug carriers. Exosomes have been shown to possess substantial benefits in targeted drug and biomolecule delivery for various diseases [114–116], making them excellent candidates for treating SLE and other autoimmune diseases. While exosomes show excellent potential as drug carriers, they also have limitations, including a lack of highly sensitive exosome detection methods and standardized extraction and purification methods and difficulties in actively adding protein molecules into exosomes. Exosome research is in its infancy, and much work remains to be done. Nevertheless, a better understanding of exosome biology and function will increase their applicability as drug carriers for treating human diseases.

## Figures and Tables

**Table 1:** Exosomal biomarkers in SLE.

Biomarker	Expression	Source	Role and function	References
NEAT1	High	Monocytes	Promotes SLE by activating Th2 cells	[117, 118]
GAS5	Low	PBMCs	Suppress SLE by inhibiting CD4^+^ T cell activation	[119, 120]
S100A4	High	Plasma	Prolongs the survival time of CD8^+^ T cells	[89, 121]
BPI	High	Exosomes	Inhibits Treg differentiation to promote SLE	[122]
ApoE	High	PBMCs	Increase the risk of SLE	[123, 124]
miR-124	Low	Serum	Suppress CD4^+^ T cells to inhibit SLE	[123, 124]
Hsa_circ_0000479	High	PBMCs	Adjust SLE progression by regulating the Wnt signaling pathway	[125, 126]

Note: *NEAT1*: nuclear paraspeckle assembly transcript 1; *GAS5*: growth arrest-specific transcript 5; *BPI*: bactericidal/permeability-increasing protein; *ApoE*: apolipoprotein E; PBMCs: peripheral blood mononuclear cells.

## Data Availability

The data used to support the findings of this study are included within the article.

## Abbreviations

SLE: Systemic lupus erythematosus
EVs: Extracellular vesicles
MV: Microvesicles
APBs: Apoptotic bodies
DCs: Dendritic cells
ESE: Early-sorting endosome
LSE: Late-sorting endosome
MVB: Multivesicular body
ApoE: Apolipoprotein E
HCC: Hepatocellular carcinoma
miRNAs: MicroRNAs
ILV: Intraluminal vesicles
LN: Lupus nephritis
MSCs: Mesenchymal stem cells
AGAP2: ArfGAP with GTPase domain ankyrin repeat and PH domain 2
Treg cells: T regulatory cells
METS: Metabolic syndrome
RAS: Renal artery stenosis
APCs: Antigen-presenting cells
IL-10: Interleukin-10
IFN: Interferon
pDCs: Plasmacytoid dendritic cells
HMH: Highly metastatic hepatocellular carcinoma
BBB: Blood-brain barrier
BCSFB: Blood-cerebrospinal fluid barrier
HCQ: Hydroxychloroquine
GC: Glucocorticoids
RTX: Rituximab
TNA: Therapeutic nucleic acids
BSP: Betamethasone sodium phosphate
AF647: Alexa Fluor 647
lncRNAs: Long noncoding RNAs
circRNAs: Circular RNAs
BPI: Bactericidal permeability-increasing protein
Tfh: T follicular helper
MAPK: Mitogen-activated protein kinase
TGF*β*: Transforming growth factor *β*
Th: T helper
TLR7: Toll-like receptor 7
C3: Component 3
C4: Component 4
TNF*α*: Tumor necrosis factor *α*
Akt: Protein kinase B
tsRNA: Transfer RNA- (tRNA-) derived small RNA
pDNA: Plasmid DNA.
